# Short-term changes in retinal and choroidal relative flow volume after anti-VEGF treatment for neovascular age-related macular degeneration

**DOI:** 10.1038/s41598-021-03179-x

**Published:** 2021-12-09

**Authors:** Giacomo Calzetti, Paolo Mora, Enrico Borrelli, Riccardo Sacconi, Guido Ricciotti, Arturo Carta, Stefano Gandolfi, Giuseppe Querques

**Affiliations:** 1 Department of Ophthalmology, University Hospital of Parma, Parma, Italy; 2 Institute of Molecular and Clinical Ophthalmology Basel, Basel, Switzerland; 3 School of Medicine, Vita-Salute San Raffaele University, Milan, Italy; 4 Ophthalmology Unit, Division of Head and Neck, IRCCS San Raffaele Scientific Institute, Milan, Italy

**Keywords:** Medical imaging, Eye diseases

## Abstract

The effects of anti-vascular endothelial growth factor (anti-VEGF) agents on the native ocular vasculature are poorly understood. This pilot study aimed to assess short-term changes in retinal and choroidal perfusion after anti-VEGF treatment for neovascular exudative age-related macular degeneration (nAMD) using the relative flow volume (RFV) parameter derived from laser speckle flowgraphy. Ten treatment-naïve nAMD patients underwent measurements of mean, maximum, minimum, and differential RFV within a retinal arteriolar segment and a choroidal vessel segment outside the neovascular area. Measurement of retinal RFV (rRFV), choroidal RFV (cRFV), and subfoveal choroidal thickness (SCT) was repeated 9 and 35 days after a single anti-VEGF injection. The treatment caused a statistically significant decrease in the mean rRFV, mean cRFV, and SCT during the follow-up (*p* < 0.05). At the intermediate visit, the mean cRFV and SCT were − 17.6% and − 6.4% compared to baseline, respectively. However, at the final measurement, the mean cRFV was not different from the baseline value, which indicated waning of the anti-VEGF effect. In conclusion, a single anti-VEGF injection in treatment-naïve nAMD resulted in a decrease in retinal arteriolar and choroidal perfusion, according to the RFV parameter, which is a promising tool to simultaneously assess retinal and choroidal perfusion changes in response to anti-VEGF therapy.

## Introduction

Age-related macular degeneration (AMD) is a leading cause of blindness worldwide^1^. The neovascular form of AMD (nAMD) is characterized by macular neovascularization (MNV), an abnormal growth of blood vessels that generally originates from the choroid and often leads to irreversible photoreceptor damage and visual loss. Anti-vascular endothelial growth factor (anti-VEGF) therapy has become the standard of care in the treatment of MNV^2^. While beneficial in the treatment of MNV, anti-VEGF agents have certain side effects, including decreased retinal cell survival and damage to the native ocular vasculature^3^. The latter effect may be due to decreased nitric oxide (NO) levels caused by VEGF suppression^4,5^. In the past decade, studies have analyzed ocular blood flow changes after intravitreal anti-VEGF treatment, but the results have been contradictory, partly because of the differences in measurement techniques and study designs^4–22^. A few studies have evaluated the effects of anti-VEGF therapy on the retinal and choroidal blood circulation simultaneously in nAMD eyes^19,20^. Laser speckle flowgraphy (LSFG) is a non-invasive technique used to simultaneously measure the blood flow in different ocular vascular beds^23–28^, and is suitable to investigate the effects of anti-VEGF treatment on the native retinal and choroidal vasculature^29^. Although the technique lacks depth resolution, it allows selective measurement of retinal and choroidal blood flow using the relative flow volume (RFV) parameter, and has high reproducibility and consistency compared to other in vitro and in vivo techniques^30–32^. This pilot study aimed to quantitatively assess the short-term effects of intravitreal anti-VEGF on the retinal and choroidal blood flow of nAMD eyes using the RFV parameter.

## Results

Ten eyes of 10 patients (3 males, 7 females; mean age: 77 ± 6 years) were selected based on the inclusion criteria. There were seven type-1 MNVs and three mixed-type MNVs. Eight patients received bevacizumab 1.25 mg/0.05 mL, while two received ranibizumab 0.5 mg/0.05 mL. The clinical characteristics of the participants are summarized in Table 1. The mean, maximum (max), minimum (min), and differential (diff) retinal RFV (rRFV) and choroidal RFV (cRFV), along with the subfoveal choroidal thickness (SCT) are shown in Table 2. Table 2 also shows the values of best-corrected visual acuity (BCVA) (mean ± standard deviation) at the considered time-points.

**Table 1:** Demographics and baseline clinical characteristics of participants. Data are shown as mean ± standard deviation.

Age (years)	77.5 ± 5.6
Sex (F/M)	7/3
Best-corrected visual acuity (logMAR)	0.50 ± 0.23
Intraocular pressure (mmHg)	15.4 ± 3.0
Systolic blood pressure (mmHg)	150.6 ± 22.1
Diastolic blood pressure (mmHg)	89.4 ± 10.8
Intake of antihypertensive agents (yes/no)	6/4

**Table 2:** Average values of study parameters. Data are shown as mean ± standard deviation.

	Baseline	Intermediate	Final	*p*
Mean rRFV (AU)	245.6 ± 80.9	225 ± 74.8	211.7 ± 83.3	0.044^‡^
Max rRFV (AU)	374.4 ± 105.2	314.4 ± 83.4	317.5 ± 106.5	0.012*^,^^‡^
Min rRFV (AU)	170.4 ± 75.3	154.9 ± 73.4	146.8 ± 78.7	0.051
Diff rRFV (AU)	204 ± 57	159.5 ± 43.3	170.7 ± 51.3	0.032*
Mean cRFV (AU)	243.9 ± 131.7	200.9 ± 110.8	226 ± 134.3	0.025*
Max cRFV (AU)	332.2 ± 180.3	266.7 ± 142.1	302.4 ± 171.9	0.042*
Min cRFV (AU)	204.1 ± 111	161.7 ± 93.1	178.7 ± 110.5	< 0.01*^,^^‡^
Diff cRFV (AU)	128.1 ± 72.8	105.1 ± 54.2	123.7 ± 62.8	0.204
BCVA (logMAR)	0.50 ± 0.23	0.49 ± 0.20	0.55 ± 0.21	0.940
SCT (µm)	202.9 ± 86.3	190 ± 83.2	191.7 ± 87.5	< 0.01*^,^^‡^

*rRFV* retinal arterial relative flow volume, *cRFV* choroidal relative flow volume, *BCVA* best-corrected visual acuity, *logMAR* logarithm of the minimum angle of resolution, *SCT* subfoveal choroidal thickness.

Data are presented as means ± SD.

Changes over time were analyzed with repeated measures ANOVA.

*Statistically significant difference between baseline and intermediate visit.

^‡^Statistically significant difference between baseline and final visit.

From baseline to the intermediate measurement, there was a significant decrease in the max and diff rRFV (*p* < 0.01 and *p* = 0.036, respectively), mean, max, and min cRFV (*p* = 0.025, *p* = 0.043, and *p* = 0.011, respectively), and SCT (*p* < 0.01).

From baseline to the final measurement, there was a significant decrease in the mean and max rRFV (*p* = 0.042 and *p* = 0.033, respectively), min cRFV (*p* = 0.022), and SCT (*p* = 0.01).

From intermediate to final measurement, the mean rRFV tended to decrease (*p* = 0.187), while the mean cRFV tended to return to the baseline value (*p* = 0.096), but no statistically significant difference was seen in any parameter.

The min rRFV and diff cRFV remained unchanged.

The mean cRFV and SCT changed in the same direction, but the decrease was more pronounced in the former (change at the intermediate visit: mean cRFV − 17.6%; SCT − 6.4%), while the correlation between the two parameters was not significant (β =  − 0.008, *p* = 0.95).

## Discussion

We found that a single anti-VEGF intravitreal injection significantly decreased retinal-arteriolar and cRFV in the parapapillary region of eyes with nAMD. Previous studies have investigated the short-term effects of anti-VEGF on the native ocular vasculature of AMD patients using different techniques. A majority of these studies used color Doppler imaging (CDI) and most of them found decreased blood-flow velocities within retrobulbar arteries and the central retinal artery shortly after anti-VEGF therapy^7,8,13^. If vasoconstriction within ciliary arteries and the central retinal artery is assumed, both blood velocity and flow, as measured in the parapapillary region, would be expected to decline because of an increase in the upstream vascular resistance. This would be in accordance with our findings of decreased retinal and cRFV. Retinal arterial vasoconstriction following ranibizumab or bevacizumab administration was demonstrated in studies using the Retinal Vessel Analyzer^6^ and bidirectional laser Doppler velocimetry (LDV)^4^, but was disproven by another study using digital fundus photography^9^. An LDV study reported a progressive decrease in retinal arteriolar blood flow at 1 and 5 weeks after bevacizumab injection^4^. We found a comparable time course of rRFV despite a higher percent decrease. More precisely, we found a 13.5% decrease over a mean of 5 weeks, which was between the 6.4% decrease of the above-mentioned study and the 17.3% decrease reported by another LDV study^10^.

Few studies have investigated the short-term changes in choroidal blood flow following anti-VEGF, mostly because of the reduced accessibility to optical techniques due to light scattering in the retinal pigment epithelium (RPE)^5,17^. Optical coherence tomography angiography (OCT-A) has emerged as a clinically useful tool to evaluate MNV^33^, and allows high-resolution vascular imaging and the simultaneous assessment of retinal and choroidal circulatory changes in response to anti-VEGF agents. A reduction in the vascular densities of both deep retinal and choriocapillaris plexus was seen in patients with a long-term history of ranibizumab or aflibercept therapy^19,21^. In these patients, increased oxygen within the inner retina due to loss of RPE and photoreceptors may cause vasoconstriction of retinal vasculature. However, quantification of blood flow using OCT-A is difficult^34^, while LSFG provides a more direct measure of perfusion^35,36^. The parameter RFV was introduced by Shiga et al. to overcome the lack of depth resolution of LSFG by separating the signal of a large retinal vessel from the background mean blur rate (MBR) originating from the underlying choroid^30^. Studies have demonstrated a favorable correlation between RFV and LDV or Doppler OCT (D-OCT)-based absolute blood flow readings in the human retina^30,31^. A recent study extended the use of RFV to mid- and large-sized choroidal vessels^32^. The study pointed out some weaknesses of the method, including the fact that choroidal vessels cannot be identified on the LSFG map in some subjects. Choroidal vessel detection is likely influenced by the absorption of the 830-nm wavelength by melanin. Therefore, measurement accuracy may be higher in patients with a relative loss of macular pigmentation, as in the case of AMD. Additionally, correlations between RFV and absolute flow did not seem to be linear over the entire physiologic flow range, with a saturation level at approximately 700 arbitrary units (AU) and an unclear relation at low flow rates^31^. This may limit comparison between vessels with different blood flow. However, retinal arterioles and choroidal vessels had comparable baseline RFV in our sample.

Another finding of the present study of note was the different durations of the anti-VEGF effects between the two vascular beds: choroidal blood flow tended to recover to baseline at the final measurement, while retinal blood flow decreased continuously. The different time courses might be due to a more prolonged effect in the retina because of its proximity to the vitreous.

The changes in cRFV and CT were in the same direction, but a correlation between the two parameters could not be established. This may be due to the different measurement sites, as cRFV was measured in the parapapillary region, whereas CT was measured in the subfoveal region. Future studies should assess the correlation between cRFV and CT using co-localized measurements. Furthermore, SCT was measured using spectral-domain OCT without enhanced depth imaging (EDI) (i.e., dedicated choroidal imaging scans), but a swept-source device may provide a more accurate measurement because of its higher wavelength.

Important limitations of the study include the small sample size and use of different anti-VEGF agents, particularly as these agents may differ in strength and secondary actions, and in molecular weights (which could affect diffusion into the choroid). Second, the study did not include a control group. Although a true control is not feasible due to ethical concerns regarding non-treatment of nAMD, patients with similar pathologies (e.g., intermediate AMD without neovascularization) could be used as controls. This could be useful to determine whether the changes seen are related to the therapy or to random measurement errors. Third, we did not evaluate the correlations with visual function or other relevant biomarkers, such as those provided by OCT-A, and did not measure MBR of the neovascular area, which was proposed as a potential biomarker of MNV perfusion^37^. Lastly, it is known that intravitreal injections have a direct effect on intraocular pressure (IOP), which could interfere with retinal or choroidal blood flow. However, we believe that the impact of IOP on the measurements was small because it tends to normalize 1 week after the anti-VEGF injection^38^.

In conclusion, the present study clearly showed a short-term decrease in the RFV of the retina and extra-neovascular choroid after a single intravitreal anti-VEGF injection in treatment-naïve patients. It is known that a reduction of blood flow may play a role in the atrophic forms of AMD, as well as in other eye diseases, such as optic neuropathy^39–41^. This pilot study presents a new tool to quantify choroidal and retinal blood flow changes after anti-VEGF, which may provide useful information about side effects and thus improve patient care. Large prospective studies with longer follow-up periods are needed to determine the clinical importance of reduced retinal and choroidal perfusion following anti-VEGF treatment.

## Methods

### Patients

The aim of this pilot study was to assess the short-term changes induced by anti-VEGF treatment on the LSFG-derived parameter RFV, measured in the retina and choroid of eyes with nAMD. Treatment-naïve exudative nAMD patients seen at the Ophthalmology Unit of the University Hospital of Parma, Italy, were included. They underwent a baseline ophthalmological evaluation, including BCVA assessment with the standard Early Treatment of Diabetic Retinopathy Study chart; dilated fundus examination; macular OCT using the Cirrus HD-OCT 4000 device (Carl Zeiss Meditec, Jena, Germany)^42^; fluorescein angiography (FA) and indocyanine green angiography (ICGA) using the Heidelberg Spectralis device (Heidelberg Engineering, Heidelberg, Germany); color fundus photography; and LSFG (RetFlow; Nidek Co. Ltd., Aichi, Japan). The participants received a loading dose consisting of three intravitreal anti-VEGF injections. The first injection was performed within 6 days of the baseline examination. Two follow-up examinations, including BCVA, dilated fundus assessment, OCT, and LSFG, were undertaken 9 ± 3 days (intermediate examination) and 35 ± 7 days (final examination) after the first injection. The second intravitreal injection was performed immediately after the final examination on the same day, while the third injection was performed 4 weeks later.

The exclusion criteria were MNV type III; polypoidal choroidal vasculopathy; other ophthalmic diseases, such as optic neuropathy; refractive error ≥ 6 diopters; significant opacity of the ocular media; any ocular surgery in the previous 6 months; and a history of intravitreal anti-VEGF therapy.

The study was approved by the Ethics Committee of the University of Parma. Written informed consent was obtained from the patients, and all procedures were performed in accordance with the Declaration of Helsinki.

### Laser speckle flowgraphy

**Figure 1:**
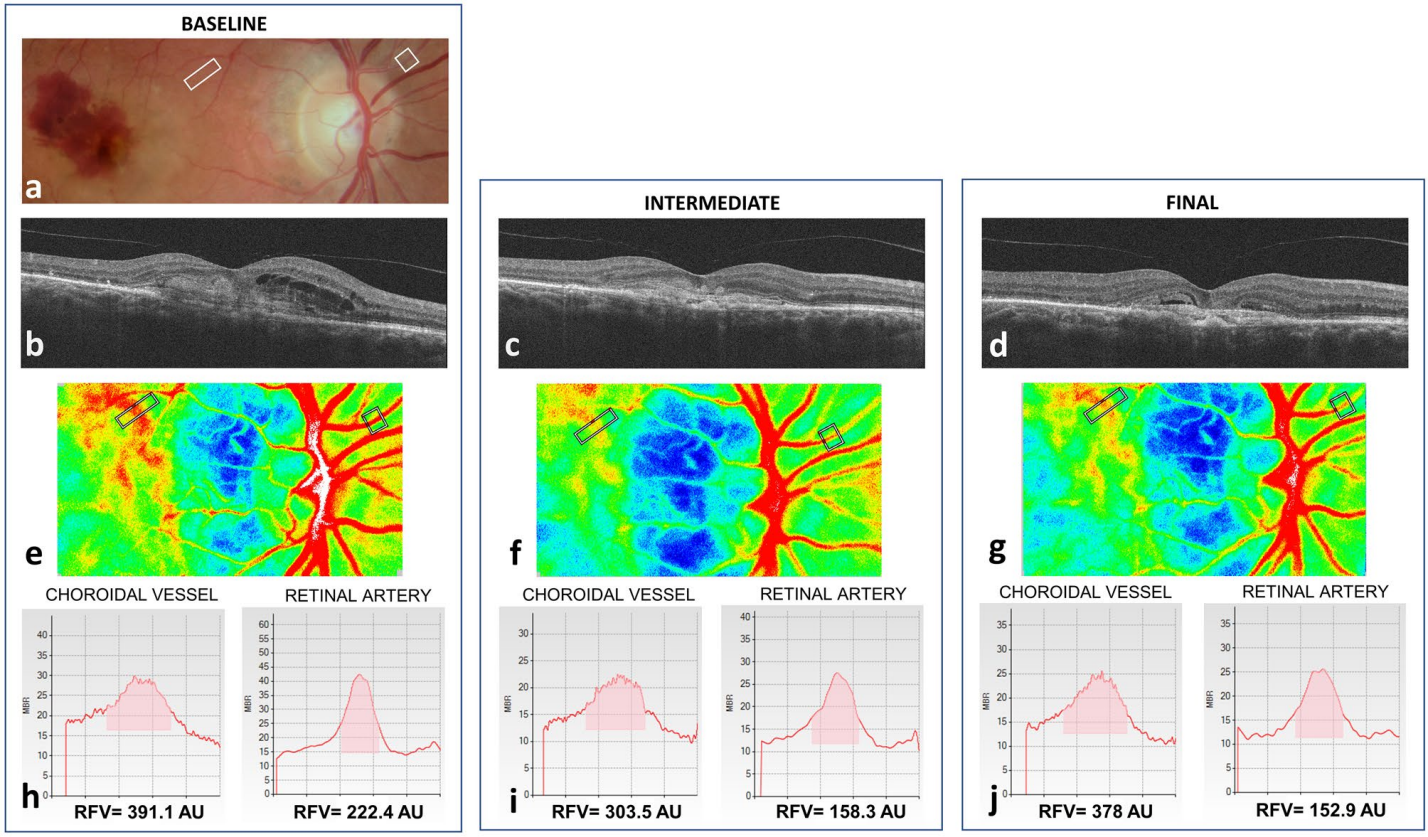
Laser speckle flowgraphy analysis. (**a**) Fundus photograph taken at baseline showing the rectangular regions of interest (ROI) used to analyze retinal and choroidal blood flow with LSFG. (**b**–**d**) Spectral-domain OCT showing the macular neovascularization before and after anti-VEGF treatment. (**e**–**g**) LSFG composite map showing the ROI used to analyze retinal and choroidal blood flow before and after anti-VEGF treatment. The ROI correspond to those shown in (**a**). (**h**–**j**) Quantitative blood flow analysis within the vessel segment of interest before and after anti-VEGF. In each graph, RFV (pink area) is obtained after subtraction of background Mean Blur Rate (MBR).

The principles of LSFG have been described in detail by Sugiyama^23^. Briefly, a random interference effect, known as the ‘speckle pattern’, is generated when a laser light illuminates a surface, such as the retina. The main LSFG index, i.e., the MBR expressed in AU, is calculated based on the changes in speckle pattern contrast caused by the movement of blood cells within ocular vessels. The commercially available LSFG RetFlow system was used in this study. This device consists of a fundus camera supplied with an 830-nm diode laser and a digital charge-coupled device (CCD) camera. The observation field was 6 × 3.8 mm at the retina. The device was equipped with an eye-tracking system and targets for internal and external fixation. A single LSFG scan consisted of 118 images, captured at a rate of 30 Hz for approximately 4 s. By averaging the 118 frames, the in-built LSFG Analyzer software (version: 1.0.0.0) produced a composite map showing the average MBR in a pseudo color or grayscale two-dimensional image (Fig. 1). Additionally, the “Heartbeat Data” analysis provided automatic measurements of the max, min, and diff MBR within the cardiac cycle.

### LSFG protocol

Patients were asked to abstain from coffee, tea, and alcohol on measurement days^43,44^. Before LSFG, the pupils were dilated using one drop of tropicamide 1%. Two consecutive LSFG scans centered on the optic nerve head were obtained by an experienced operator in a dim room. All scans passed the binary quality control of the instrument. Shortly after LSFG, IOP (obtained using Goldmann applanation tonometry), and systolic and diastolic blood pressure (obtained using a sphygmomanometer) were measured in the seated position. At follow-up examinations, the ‘Follow-up’ function of the RetFlow was used to rescan the same region with the same laser beam intensity.

### LSFG analysis

The in-built LSFG analyzer software (version: 1.0.0.0) was used for the analysis. In each baseline composite map, a major retinal artery and choroidal vessel were measured in the parapapillary region using a rectangular region of interest, as previously described (Fig. 1)^30,32^. Vessels of interest were checked on fundus photography and angiography to confirm the location outside of the MNV area, and the absence of overlying hemorrhage and lipid exudation. The signal arising from the vessel segment of interest was automatically separated from the background MBR by computing a threshold between the MBR values of the vessel segment and microvasculature on either side of the vessel segment. The method assumes that background MBR could be approximated using the MBR of the microvasculature adjacent to the vessel segment. The software then extracts the mean, max, and min RFV in AU, as a measure of blood flow in the vessel segment of interest. Diff RFV was calculated as [max RFV − min RFV]. An experienced operator checked all frames for the presence of artifacts (e.g., floaters overlying the region of interest and eye tracking errors), and the frames with artifacts were discarded. The discarded frames comprised less than 50% of all frames in all scans. The means of the two consecutive scans were taken. Baseline regions of interest (ROIs) were saved and exported to the follow-up LSFG scans. Although the same ROI can automatically be repositioned in the same location in the retina, manual adjustment ensured the accuracy of ROI positioning on follow-up scans.

### OCT analysis

SCT was manually measured on an HD raster scan using the caliper tool of ImageJ software (National Institutes of Health, Bethesda, MD, USA) by two experienced graders (EB and RS), as previously described^45^. Continuity of the OCT scan position was achieved using vascular landmarks of the baseline scan as a reference for follow-up scan acquisition and analysis.

### Statistical analysis

The Shapiro–Wilk test was used to assess the normality of the data distribution and did not show any statistically significant deviations (*p* > 0.19 for all parameters). Continuous variables are expressed as means ± standard deviation (SD). BCVA data were converted to logarithm of the minimum angle of resolution (logMAR) units. Serial data were analyzed using repeated measures analysis of variance (ANOVA), followed by Fisher’s least significant difference test. In repeated-measures ANOVA tests, sphericity was not assumed and the Geisser–Greenhouse correction was applied. The correlation between cRFV and SCT was analyzed using generalized estimating equations considering the repeated measures. A *p*-value < 0.05 was considered statistically significant for all analyses. Data were analyzed using SPSS Statistics software (version: 25.0; IBM Corp., Armonk, NY, USA) and GraphPad Prism 9.0.1 (GraphPad Software, Inc., La Jolla, CA, USA).

### Ethics approval

Approval was obtained from the ethics committee of University of Parma. The procedures used in this study adhere to the tenets of the Declaration of Helsinki.

## Data Availability

The datasets analyzed in the current study are available from the corresponding author on request.
